# ASSESSMENT OF RISK OF FALL IN THE HOME ENVIRONMENT OF KOREAN OLDER ADULTS: REVISION AND VALIDATION OF THE K-HOME FAST

**DOI:** 10.1093/geroni/igae098.2787

**Published:** 2024-12-31

**Authors:** Min Kyung Park, Gwang Suk Kim, Layoung Kim, Jae Jun Lee, Namhee Kim, Ji Yeon Lee

**Affiliations:** Mo-Im Kim Nursing Research Institute, Yonsei University College of Nursing, Seoul, Republic of Korea; Yonsei University, Seoul, Republic of Korea; Yonsei University, Seoul, Republic of Korea; Yonsei University, Seoul, Republic of Korea; Yonsei University, Seoul, Republic of Korea; Inha University, Incheon, Republic of Korea

## Abstract

Most older adults experience a fall inside their house. Because the home environment varies based on culture and time, the tools to assess fall risk must be regularly modified and validated. Therefore, this study aimed to develop the Revised K-HOME FAST (Korean version of the Home Falls and Accidents Screening Tool) and evaluate its validity and reliability. The Revised K-HOME FAST was developed in these phases: 1) development of the Revised K-HOME FAST, 2) feasibility test, 3) verification of content validity, and 4) data collection and verification of reliability. Internal consistency was confirmed by conducting home-visit assessments with 211 community-dwelling older adults who had experienced at least one fall in the local community. To verify inter-rater reliability, six investigators were paired and independently assessed the homes of 20 older adults. The final Revised K-HOME FAST consisted of 25 items categorized into seven home areas: bathroom/toilet, bedroom, living room, kitchen, stairs, entrance, and entire house. The item-content validity index of the preliminary version of Revised K-HOME FAST ranged from 0.8 to 1.0, except for one item. The internal consistency (Kuder–Richardson Formula 20) of the Revised K-HOME FAST was.65. The agreement percentage for each item ranged from 92% to 100%, and the intraclass correlation coefficient ranged from 0.79 to 1.00, indicating high inter-rater reliability. The Revised K-HOME FAST has satisfactory validity and reliability for assessing the risk of falls in the home environment of community-dwelling older adults in Korea.

